# Topology Optimization of Passive Cell Traps

**DOI:** 10.3390/mi12070809

**Published:** 2021-07-09

**Authors:** Zhiqi Wang, Yuchen Guo, Eddie Wadbro, Zhenyu Liu

**Affiliations:** 1 Changchun Institute of Optics, Fine Mechanics and Physics (CIOMP), Chinese Academy of Sciences, Changchun 130033, China; wangzhiqi18@mails.ucas.ac.cn; 2 School of Optoelectronics, University of Chinese Academy of Sciences, Beijing 100049, China; 3 Ji Hua Laboratory, Foshan 528000, China; guoych@jihualab.com; 4 Department of Mathematics and Computer Science, Karlstad University, SE-651 88 Karlstad, Sweden; eddie.wadbro@kau.se

**Keywords:** topology optimization, cell capture, flow distribution, periodic layout

## Abstract

This paper discusses a flexible design method of cell traps based on the topology optimization of fluidic flows. Being different from the traditional method, this method obtains the periodic layout of the cell traps according to the cell trapping requirements by proposing a topology optimization model. Additionally, it satisfies the cell trapping function by restricting the flow distribution while taking into account the overall energy dissipation of the flow field. The dependence on the experience of the designer is reduced when this method is used to design a cell trap with acceptable trapping performance. By comparing the influence of the changes of various parameters on the optimization results, the flexibility of the topology optimization method for cell trap structure optimization is verified. The capability of this design method is validated by several performed comparisons between the obtained layouts and optimized designs in the published literature.

## 1. Introduction

Microfluidic chips have the advantages of high efficiency and low cost in analytical chemistry and biological research. The technology has developed rapidly during the past 20 years and is today applied to gene sequencing, cell detection and culture, and sample preparation and analysis. Cell analysis and detection are important branches of biology and clinical medicine and are of great significance to the diagnosis and treatment of related diseases.

Single-cell analysis can more accurately understand the differences of individual cells to better understand diseases, such as cancer. Efficient single-cell capture is the basic requirement of single-cell analysis, and only efficient capture can provide valuable information for the clinic. In 2002, Thorsen et al. [1] first proposed the development of parallel sample and cell processing systems. Since then, different methods of capturing cells have become the basis for studying single-cell responses. Traditionally, the serial dilution method is used for single-cell analysis. The cell sample is diluted to a concentration of only one cell in hundreds of microliters, and then the single cell is placed on a conventional platform, such as a 96-well plate. However, this method’s capture efficiency is very low, so it is rarely applied. On the other hand, microfluidic technology can provide high-throughput unit operations and can be controlled precisely at the unit level.

The microfluidic chip provides a platform for analyzing single cells by combining active or passive single-cell capture methods. Active single-cell capture methods, such as dielectric electric [2,3,4,5], optical [6,7,8,9], magnetic [10,11,12,13,14], or acoustic field [15,16,17], can provide precise cell manipulation and selective cell capture. However, these methods require external devices and need precise control. In contrast, passive capture methods using fluid flow force are more advantageous because they are simple to implement, do not require any external equipment and professional technology to control, and only need to give the microfluid a certain power to make it flow.

In a microfluidic system, the most common way to achieve cell or particle capture is to create a relatively small side channel in the main transport channel. When a part of the total flow is sucked into the side channel, cells bigger than the side channel may become stuck at the side channel’s entrance. An obvious advantage of this method is that the release function can also be achieved when the channel flow is reversed. To achieve sequential capture and large-scale parallel screening of single cells, Tan and Takeuchi [18] proposed a flow device in which the resistance of the capture flow channel is much smaller than that of the main flow channel. The treatment of alginic acid microspheres encapsulated cells shows its potential in cell screening [19]. The method of single-cell capture in array format was also applied by Lee et al. [20]; he designed a pinball-style hydrodynamic capture array, in which the capture column array is arranged obliquely in a flow chamber, and each capture column is designed with a groove used to capture single cells or smaller groups of cells. The concept of this capture array was further developed by Skelley et al. [21], which can achieve not only single-cell capture but also a cell paired capture array at the appropriate depth. In 2011, the hydrodynamic guided single-cell capture array proposed by Chung [22] achieved 80% capture efficiency and allowed time-lapse monitoring of the behavior of single cells.

The flow cell capture method uses fluid flow to guide cells into the trap position and captures the cells at a relatively fixed position for detection. The main flow direction can be the same as the capture direction [23] or perpendicular [24,25]. The capture efficiency mainly depends on the flow resistance of the whole basin and the difference in flow resistance between the capture channel and the bypass flow channel. The traditional structural design method cannot quantitatively control the flow ratio between the capture channel and the bypass channel and cannot guarantee high capture efficiency. Moreover, it only pays attention to the relative difference of the flow resistance of the two flow channels and does not consider the flow resistance of the overall basin. The manual shape optimization method strongly relies on the designers’ experience. Here, we present an automated, efficient, and practical structural design method for passive cell traps.

The core of the cell trap design is the design of the flow channel structure (including the capture flow channel and the bypass flow channel). This paper proposes a topology optimization method to optimize the structure of cell traps. The objective function is a necessary condition for the topology optimization model to be able to optimize the flow channel structure. In this paper, the energy dissipation is taken as the objective function, and the outlet flow ratio is taken as the constraint to construct a fluid topology optimization model. In addition, the fluid is generally laminar when flowing through the cell trap, and the flow channel structure obtained by using a topology optimization method targeting energy dissipation can keep the fluid flow laminar. In summary, the topology optimization model applies to the design of cell traps.

In 2003, Borrvall and Petersson [26] studied steady Stokes flow with low Reynolds number and established a basic density-based topology optimization model for fluid flow. Subsequently, Gersborg-Hansen and Sigmund et al. [27] extend the density topology optimization method to the steady Navier–Stokes flow with medium and low Reynolds numbers and considering convection effects. In 2010, Evgrafov [28] realized the topology optimization of weakly compressible fluids for the first time. For the topology optimization of unsteady flow, Kreissl et al. [29] proposed the application of discrete adjoint realization, and Deng et al. [30] derived the continuous adjoint equation [30]. The fluid topology optimization method has been applied to the design of a variety of microfluidic devices, such as Tesla valves [31,32], mixers [33,34,35], and flow distributors [36,37]. In the design of flow distribution, Liu [37] proposed a flow channel topology optimization problem with equal flow constraints, using the Lagrangian multiplier method to achieve equality constraints on the specified boundary, and using the quadratic penalty term and cut-off sensitivity to maintain optimized stability. Zhou [37] used the real-reference model, which combined the computational fluid dynamics (CFD) method with a numerical optimization method, and proposed a channel network optimization design method with user-specified outlet flow and fixed inlet drive flow as constraints.

This paper focuses on the design of cell traps based on the real-reference model for fluid topology optimization. Multi-objective optimization is carried out with flow ratio and energy dissipation as the target. Different flow ratios can be achieved by adjusting the parameters to obtain the corresponding topology and shape, which greatly improves the capture performance of the cell trap. When optimizing a device to manipulate the fluid, the cost is typically related to the energy dissipation across the component. When hundreds of cell capture cycles are arrayed sequentially, the energy dissipation throughout the capture channel determines the energy cost. For the same number of capture cycles and the same energy cost, capture structures with a lower energy dissipation generally have a higher capture efficiency. This paper provides an automated design method for cell traps. The designer only needs to change several parameters according to its size, flow ratio, and other requirements to obtain the corresponding structural shape, which reduces the designer’s experience requirements and design threshold.

This paper is organized as follows: the topology optimization model of the flow distributor is introduced in Section 2; numerical examples for capturing single cells and multiple cells when the cell capturing direction is perpendicular to the main flow direction and comparisons of optimized structures under different parameters are given in Section 3; the cell trap shape optimized in this paper is compared with the traditional method in terms of capture efficiency and energy dissipation, showing the advantages of this optimization method. Some conclusions are given in Section 4.

## 2. Materials and Methods

The flow determines the movement path of the cells in the capture unit. To achieve sequential capture, the flow resistance of each capture unit should be smaller than the flow resistance of the next unit. To capture only one cell in a capture cycle, the flow rate of the capture channel should be greater than the flow rate of the bypass channel. That is, the flow rate ratio at the two outlets is selected to satisfy:

(1) $$n_{q}=Q_{\mathrm{trap}}/Q_{\mathrm{bypass}}>1,$$

where $Q_{\mathrm{trap}}$ represents the flow rate of the capture channel in each capture unit, and $Q_{\mathrm{bypass}}$ represents the flow rate of the bypass channel. For the situation where multiple cells need to be captured in one capture cycle, the flow rate ratio of the capture channel must meet:

(2) $$n_{Q}=Q_{i}/Q_{i+1}>1,$$

here, for any integer *j*, $Q_{j}$ represents the flow rate of the *j*th capture channel in the same capture cycle.

A single-cell capture unit can be regarded as a flow distributor with one inlet and two outlets with different widths. Similarly, a multi-cell capture unit can be regarded as a flow distributor with one inlet and several outlets with different widths. The real-reference model topology optimization method of the flow distributor can be used to design the passive cell capture unit structure [37]. The real model represents a fixed inlet flow to the design area, the outlet pressure value is 0, and all other boundaries have non-slip conditions. The reference model represents the artificially set flow ratio at the outlet of the same design area, the inlet pressure is 0, and all other boundaries have non-slip conditions. Substituting the Darcy force for the physical force term in the incompressible Navier–Stokes equation to simulate the fluid motion in the design area, we get

(3) $$\begin{array}{l} \rho (u\cdot \nabla )u-\eta \nabla \cdot (\nabla u+\nabla u^{T})+\nabla p=-\alpha u,\;\mathrm{in}\;\Omega \\ \nabla \cdot u=0,\;\mathrm{in}\;\Omega \end{array}$$

where Ω is the design area, *ρ* is the fluid density, u is the fluid velocity, p is the fluid pressure, η is the fluid viscosity, α is the impermeability of the porous medium, which is defined using the interpolation function of the design variable γ:

(4) $$\alpha (\gamma )=\alpha_{\min}+(\alpha_{\max}-\alpha_{\min})\frac{q(1-\gamma )}{\left(q+\gamma \right)},$$

where $\alpha_{\max}$ and $\alpha_{\min}$ are the maximum and minimum values of α(γ), respectively, and q is a positive parameter used to adjust the convexity of the interpolation curve. The value of the design variable γ varies from 0 to 1. When γ = 0, it corresponds to the solid area, and when γ = 1, it corresponds to the liquid area.

The objective of the optimization model is to minimize

(5) $$J=\frac{\Phi_{\mathrm{real}}+\Phi_{\mathrm{ref}}}{b+v^{T}(1-\gamma )}+\theta (\Phi_{\mathrm{real}}-\Phi_{\mathrm{ref}}),$$

where Φ is the energy dissipation in the computation domain, the subscripts real and ref represent the real flow field and the reference flow field in the optimization model, the first term is in fractional form, the numerator is the sum of the energy dissipation of the two models, $v^{T}\left(1-\gamma \right)$ is the volume of the solid phase material in the domain, and b is the volume coefficient, used to adjust the appropriate structural volume. The second term is the difference in fluid energy dissipation under the two models, and θ is the weighting coefficient. In this method, the flow field in the two cases in the optimization results tends to be consistent, thereby realizing the outlet flow restriction in the real model. In the objective function, both the parameters b and θ are designer predefined scalar numbers but not spatially dependent variables. In the topology optimization model, these two parameters can regulate the detailed shape of the fluidic region in the overall design domain. The parameter b determines the ratio of the distribution of the fluid to the solid region; the larger the value of b, the larger the volume fraction of the fluid subdomain exists in the final optimized results. The parameter θ determines structure distribution in relation to the outlet flow ratio. The calculation formula of energy dissipation is:

(6) $$\Phi =\int_{\Omega }[\frac{\eta }{2}(\nabla u+\nabla u^{T}):(\nabla u+\nabla u^{T})+\alpha (\gamma )u^{2}]d\Omega .$$

We define the relative error of the flow at a single outlet as

(7) $$R_{i}^{\left(k\right)}=(Q_{i}^{\left(k\right)}/Q_{i}-1)\times 100\%,$$

where $Q_{i}^{\left(k\right)}$ is the actual flow of the *i*th outlet, and $Q_{i}$ is the artificially set flow of the *i*th outlet in the reference model. When the absolute value of R is less than 3%, it is considered that the set flow is satisfied. When R > 0, it indicates that the outlet flow is greater than the set flow, otherwise less than the set flow. In the optimization model, the parameter θ is the weight coefficient of the flow constraint and $\theta =\theta_{0}$ is set at the beginning of the optimization. Its value is gradually increased during the optimization process to ensure that the flow relative error of the optimization result is less than 3%. The specific process is: in the *k*th iteration step, if the absolute value of the relative error of the flow at a certain outlet increases compared to the previous iteration step, that is $\left|R_{i}^{\left(k\right)}\right|-\left|R_{i}^{k-1}\right|>0$, then the value of θ increases. Otherwise, the value of θ remains unchanged.

## 3. Numerical Examples

In this section, numerical examples are given for capturing single cells and multiple cells when the cell capturing direction is perpendicular to the main flow direction. In this paper, the material interpolation function α(γ) has $\alpha_{\max}=10^{10}$,$\;\alpha_{\min}=0$, q=1, the fluid density is $998\;\mathrm{kg}/m^{3}$, and the viscosity is $10^{-3}\;\mathrm{Pas}$. In practice, the cells are mostly non-spherical and deformable. However, the cells are regarded as rigid and spherical particles in the simulation in order to simplify the numerical simulation without considering the fluidic–solid coupling in this paper. Assuming that the diameter of the captured cell is 20 μm, the cell position is reserved below the design domain, which is achieved by adding a volume constraint:

(8) $$\int_{\Omega_{\mathrm{cell}}}\gamma d\Omega /\int_{\Omega_{\mathrm{cell}}}1d\Omega >0.99,$$

where $\Omega_{\mathrm{cell}}$ is a circular area with a radius of 10 μm. The initial design variables γ of the design domain are all 0.5.

The numerical examples in this section are implemented using the commercial software COMSOL Multiphysics 3.5 (COMSOL, Inc., Stockholm, Sweden) to solve the Navier–Stokes equation with finite element and analyze sensitivity, and using the MMA (The Method of Moving Asymptotes) algorithm to update the design variables [38]. During the optimization procedure, the Navier–Stokes equations and the adjoint equations are solved using bilinear approximation, which interpolates the fluidic velocity and the pressure linearly. The design variable is interpolated linearly based on the corner nodes of the elements.

### 3.1. Single-Cell Capture Model

**Figure 1:**
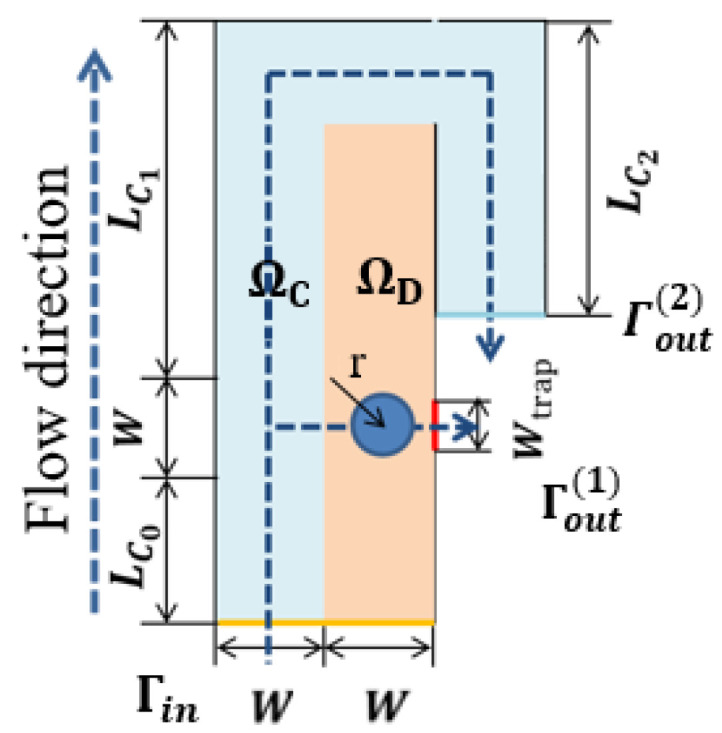
The computational domain $\Omega =\Omega_{C}\cup^{\;}\Omega_{D}$ of a single-cell capture unit with the capture channel perpendicular to the inlet channel, where $\Omega_{D}$ is the design domain, and $\Omega_{C}$ is the bypass channel.

As our first experiment, we design a single-cell capture structure. Figure 1 shows the calculation domain $\Omega =\Omega_{C}\cup^{\;}\Omega_{D}$ used for this example. Here $\Omega_{C}$ is the bypass channel, $\Omega_{D}$ is the design domain; $\Gamma_{\mathrm{in}}$ is the entrance of the capture unit, $\Gamma_{\mathrm{out}}^{\left(1\right)}$, $\Gamma_{\mathrm{out}}^{\left(2\right)}$ are the outlets of the capture channel and the bypass channel, respectively; the inlet width and the bypass outlet width *W* = 25 μm, the capture channel outlet width $W_{\mathrm{trap}}=15\;\mu m$, the flow channel length $L_{C0}=50\;\mu m$, the bypass flow runner $L_{C1}$ and $L_{C2}$ are 125 μm and 100 μm, respectively.

**Figure 2:**
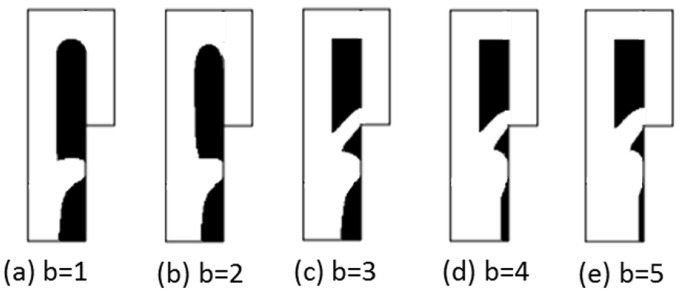
The optimization results with different volume coefficients b.

**Figure 3:**
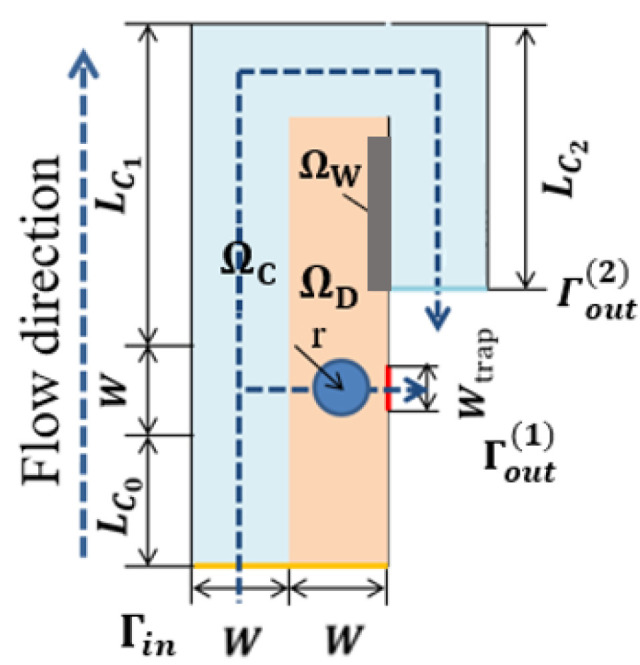
The artificial wall $\Omega_{W}$ where the design variable is forced to 0 is added to the design domain $\Omega_{D}$ of a single-cell capture unit.

As shown in Figure 2, the optimization results under different volume coefficients when the Reynolds number is 1, the flow ratio $n_{q}$ is 2, and the weight coefficient θ is 5. The white area is liquid (γ = 1), the black area is solid (γ = 0). It can be seen that b affects the distribution ratio of fluids to solids in the optimization results. The topological form of the optimization result changes when the weight coefficient is greater than or equal to 3, resulting in the new flow channel allowing the fluid to flow directly to the bypass outlet to shorten the flow distance. The new flow channel makes a predefined bypass flow path almost have no flow at the top of the fluidic channel (channel with a rectangular shape) when compared with the cases b = 1 and b = 2, causes the cells to block the newly created flow channel. To avoid this situation, as shown in Figure 3, a designated partial area $\Omega_{W}$ on the side of the design domain close to the bypass outlet is a 50 μm long and 10 μm wide non-design domain to block fluid from flowing through this area. Another way to avoid creating a new flow channel is to set the internal boundary in Figure 1 as a wall. The subsequent optimized results have no additional fluidic branch (except for the position to capture the particle) when adjusting the optimization parameters b and θ.

**Figure 4:**
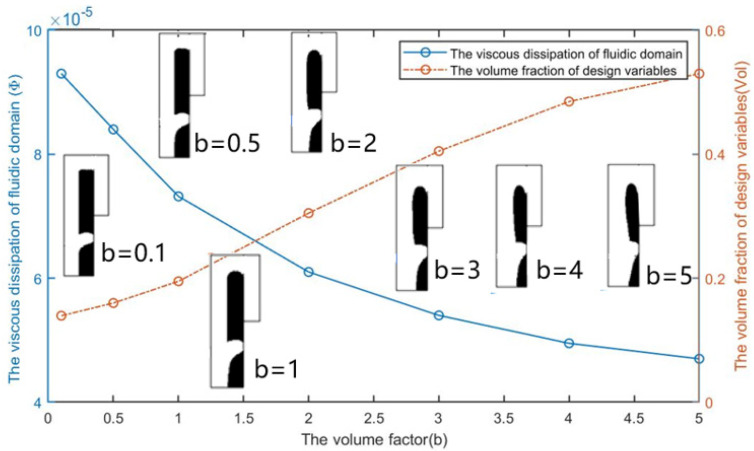
The optimization results and the effect of the volume coefficient b on the volume fraction vol of design variables and the viscous dissipation Φ of fluidic domain.

From Figure 4, we can see that the topological shape of the optimization result remains unchanged after adding the non-design area when *Re* = 1, $n_{q}$ = 2.0, and θ = 5. As the volume coefficient b increases, the volume fraction of the fluid area of the optimization result increases, and the energy dissipation of the entire fluid calculation area decreases with the volume fraction increases.

**Figure 5:**
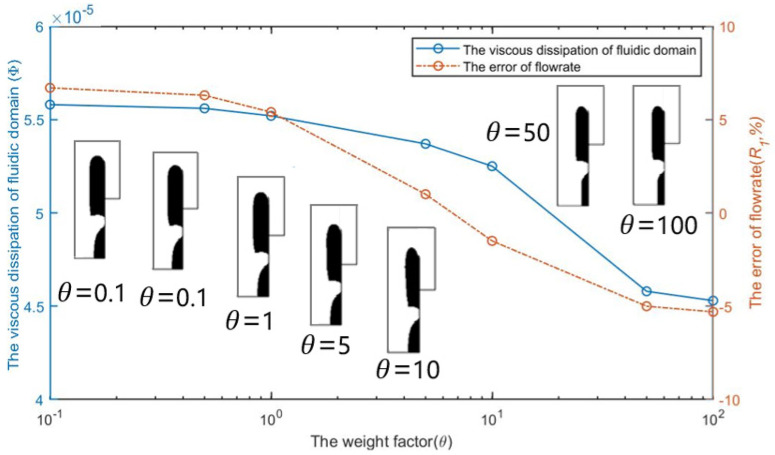
The optimization results and the effect of the weight coefficient θ on the error $R_{1}\;$ of flowrates and the viscous dissipation Φ  of fluidic domain.

Figure 5 shows the optimization results of selecting different weighting coefficients θ in the objective function and its influence on the energy dissipation in the calculation domain and the flow error of capture channel when *Re* = 1,$\;n_{q}$ = 2.0, and b = 3. It can be seen that the energy dissipation in the calculation domain and the flow error of the capture channel decrease with the increase of the weight coefficient θ. When θ>10, the actual flow of the capture channel is less than the set flow, thus excessive weighting coefficient brings difficulties to obtain the set flow ratio.

**Figure 6:**
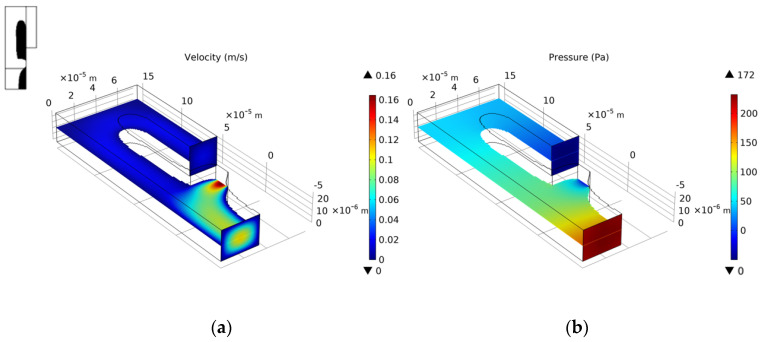
(**a**) The velocity field distribution and (**b**) pressure field distribution of 3D model without captured cell.

**Figure 7:**
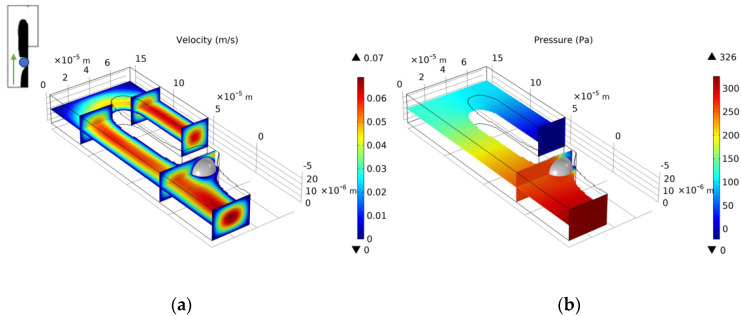
(**a**) The velocity field distribution and (**b**) pressure field distribution of 3D model with captured cell.

**Figure 8:**
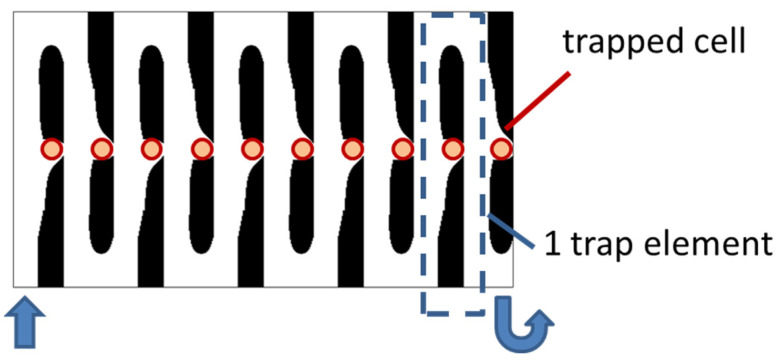
Cell capture array with capture direction perpendicular to flow direction (1 × 10).

We choose the optimization result of *Re* = 1,$\;n_{q}\;$= 2.0, b = 3, and θ = 5 to build a three-dimensional model. We set the vertical height of the three-dimensional model to 25 μm. Since the height of the capture outlet is greater than the diameter of the cell, when the cell is captured, a small part of the flow of the capture outlet still flows out on the upper and lower sides. The velocity distribution of the entrance section is consistent with the optimization model. Figure 6 shows the velocity field distribution and pressure field distribution of the three-dimensional model. Through finite element calculation, the flow ratio at the two outlets is $Q_{\mathrm{trap}}/Q_{\mathrm{bypass}}=4.816>1$, which meets the needs of cell capture. Figure 7 shows the flow field distribution and pressure field distribution of the three-dimensional model when the simulated capture channel successfully captures cells (cell diameter 20 μm). It can be observed that there is a pressure difference on both sides of the cell, which keeps the cell fixed in the capture position and from moving. By calculating the flow ratio of the two outlets at this time is $Q_{\mathrm{trap}}/Q_{\mathrm{bypass}}=0.320$. Connect the bypass outlet of the previous capture unit to the inlet of the next capture unit to obtain a cell capture unit array with a capture direction perpendicular to the flow direction, as shown in Figure 8.

### 3.2. Multi-Cell Capture Model

**Figure 9:**
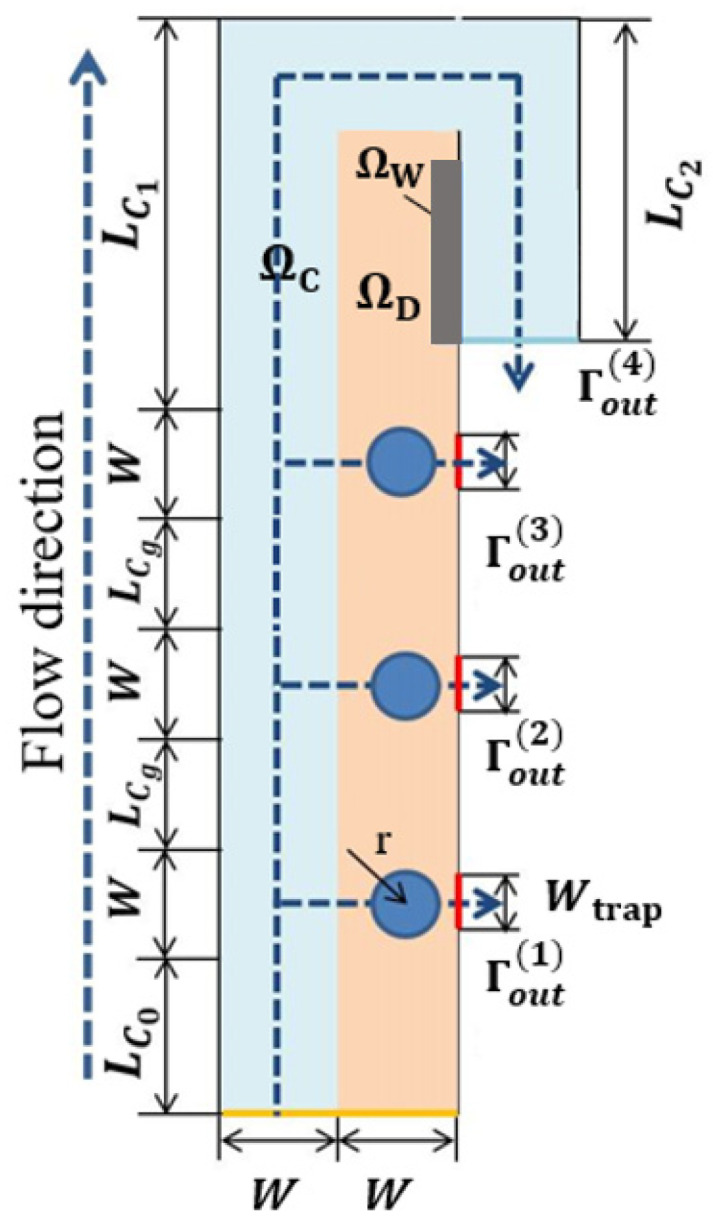
The computational domain $\Omega =\Omega_{C}\cup^{}\Omega_{D}$ of a three-cell capture unit with the capture channel perpendicular to the inlet channel, where $\Omega_{D}$ is the design domain, $\Omega_{C}$ is the bypass channel, and $\Omega_{W}$ is artificial wall.

Next, we use the topology optimization method to design a multi-cell capture unit structure. The computational domain and the design domain are shown in Figure 9, where a transition distance $L_{C_{g}}$ is added between each cell capture channel, and the length is 25 μm. All other sizes are the same as in the single-cell capture model, and the flow ratio between the outlets of each flow channel is set to 4:3:2:1, and the Reynolds number is 1.

**Figure 10:**
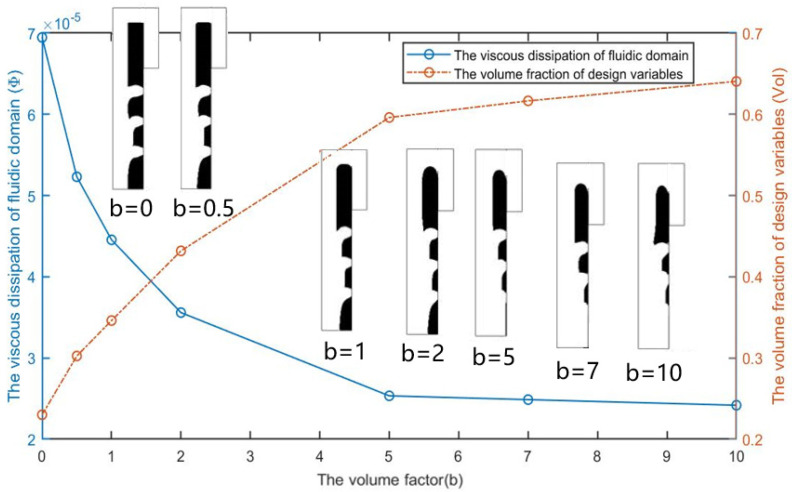
The optimization results and the effect of the volume coefficient b on the volume fraction vol of design variables and the viscous dissipation Φ  of fluidic domain.

Figure 10 shows the optimization results of the three-cell capture unit when *Re* = 1, $\;n_{Q}$ = 4:3:2:1, and θ = 50. As the volume coefficient b increases, the fluid material volume fraction of the optimization results increases, and the energy dissipation of the entire fluid calculation area decreases as the volume fraction increases. It can be observed that the value of energy dissipation is divided into two stages: when the volume coefficient b < 5, as the volume coefficient b increases, the width of the three trapping channels gradually increases, and the overall energy dissipation value of the trapping unit decreases significantly; when the volume coefficient b > 5, there is no solid material between the inlet and the first trapping channel to be optimized. At the same time, to ensure the flow rate ratio among the outlets of each channel, further increasing the volume coefficient b will not cause a large change in the volume fraction in the design domain, and the overall energy dissipation value of the capture unit is basically unchanged.

**Figure 11:**
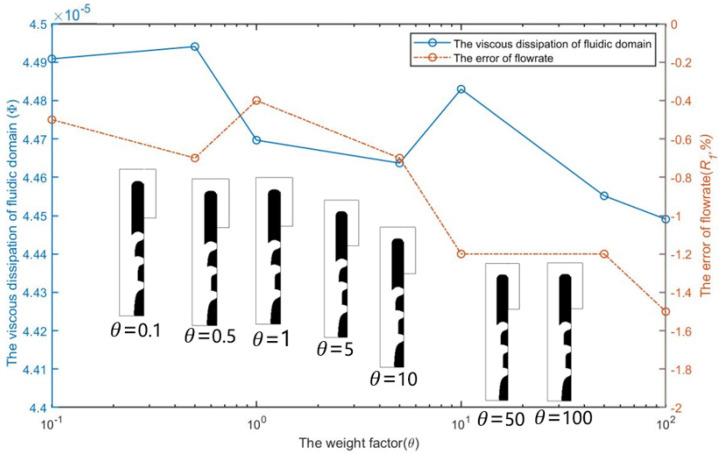
The optimization results and the effect of the weight coefficient θ on the error $R_{1}\;$ of flowrates and the viscous dissipation Φ  of fluidic domain.

Figure 11 shows the optimization results of choosing different weighting coefficients θ in the objective function and its influence on the energy dissipation in the calculation domain and the flow error of the first capture channel when *Re* = 1, $\;n_{Q}$ = 4:3:2:1, and b = 1. Different from the optimization design of a single-cell capture unit, the optimization result is not sensitive to the selection of the weight coefficient θ. After the weight coefficient θ is increased by three orders, the overall energy dissipation does not change significantly, and the flow error of the first capture channel increases by 1%.

**Figure 12:**
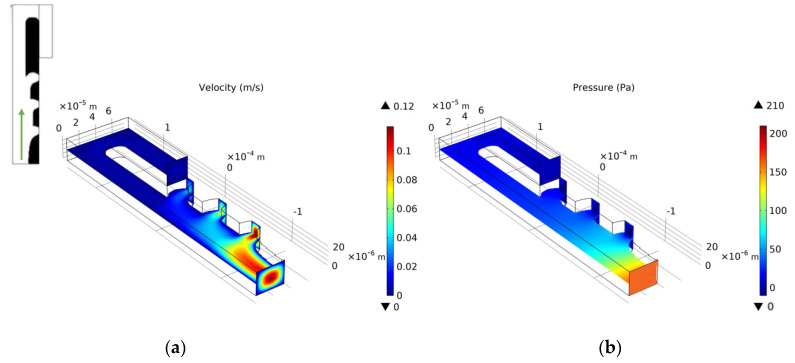
(**a**) The velocity field distribution and (**b**) pressure field distribution of 3D model without captured cell.

**Figure 13:**
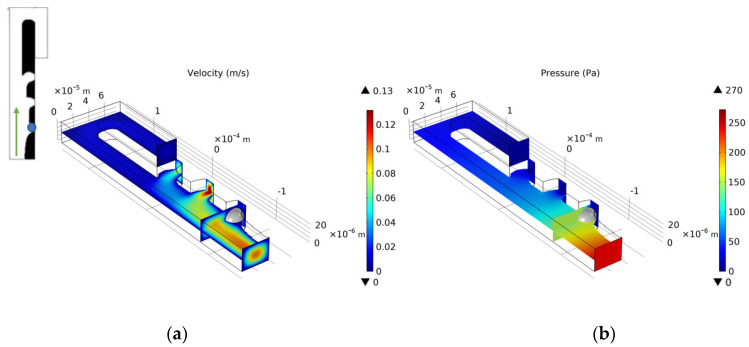
(**a**) The velocity field distribution and (**b**) pressure field distribution of 3D model with one captured cell.

**Figure 14:**
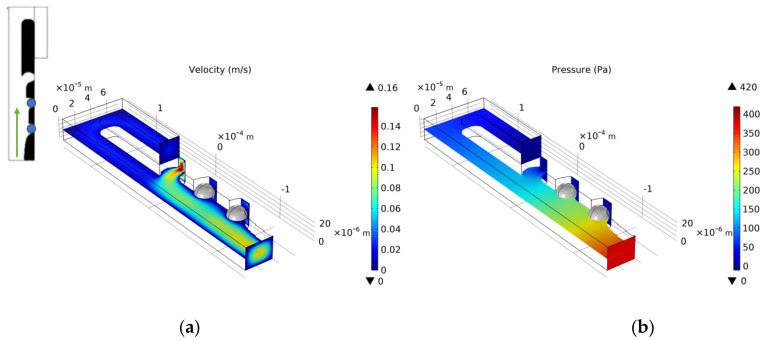
(**a**) The velocity field distribution and (**b**) pressure field distribution of 3D model with two captured cells.

**Figure 15:**
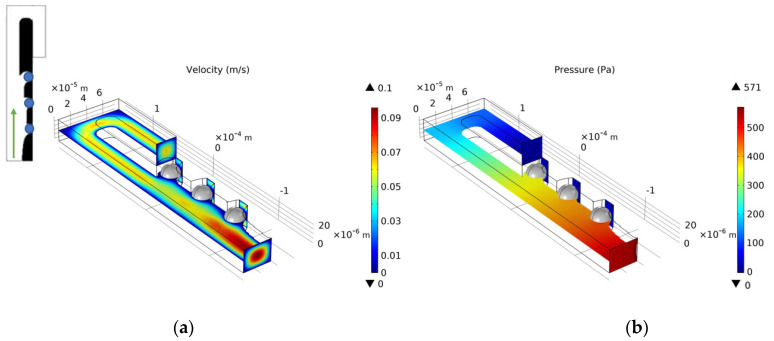
(**a**) The velocity field distribution and (**b**) pressure field distribution of 3D model with three captured cells.

**Figure 16:**
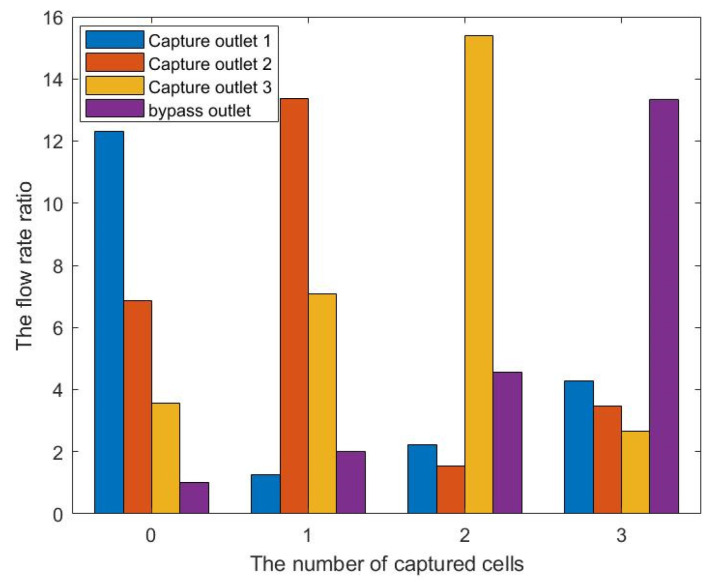
The flow rate ratio among each outlet of the three-cell capture unit and the bypass outlet under different numbers of captured cells.

We select the optimization result of volume coefficient b = 1 in Figure 10 to build a three-dimensional model with a vertical height of 25 μm. Similarly, as for the single-cell capture, after the cells are captured, the flow rate of the capture outlets are not equal to 0. The velocity distribution of the inlet horizontal section is consistent with the optimized model. Figure 12, Figure 13, Figure 14 and Figure 15 shows the velocity field distribution and pressure field distribution of the three-dimensional model when different numbers of cells are captured (*Re* = 1, $\;n_{Q}\;$= 4:3:2:1,  b = 1,  θ = 50). It can be seen that the pressure difference among the inlet and outlets increases as the number of captured cells increases. Through the finite element calculation, the flow ratios of 0 to 3 cells captured at the four outlets are obtained as: (1) 12.30:6.87:3.56:1; (2) 0.63:6.64:3.51:1; (3) 0.49:0.339:3.38:1; (4) 0.32:0.26:0.20:1, as shown in Figure 16. It can be seen that the ratio of each capture flow channel to the subsequent total flow is greater than 1, and the capture of cells has a small effect on the flow ratio among subsequent flow channels, which meets the needs of cell capture.

### 3.3. Comparison

To verify the effectiveness of the cell trap design method discussed in this paper, a comparison was made between the obtained cell trap structure using topology optimization method and the other literature results. The comparison can be made from two perspectives, (1) comparing the energy dissipation value of the fluid flow with the same capture period length and (2) comparing the outlet flow ratio of the same capture period length. In the following, we will compare based on calculation results.

In 2007, the geometrically induced flow resistance manipulation capture method proposed by Tan and Takeuchi created a competitive method for single-cell capture and was subsequently further applied to single-cell capture and analysis. Yoon [25] made further improvements to the flow capture method and proposed a further vertically restricted (FVR) geometry to avoid cell accumulation. Yang [24] designed a cell capture array consisting of an s-shaped loop channel and thousands of aligned trap units, which can be extended to a large area on a single chip to achieve high-throughput cell capture. According to the optimized shape they obtained, in the fluid area of the same size as shown in Figure 1 and under the same inlet flow rate ($3.8\times 10^{-11}\;m^{3}/s$), the flow field distribution when the capture channel does not capture the cells is calculated.

**Figure 17:**
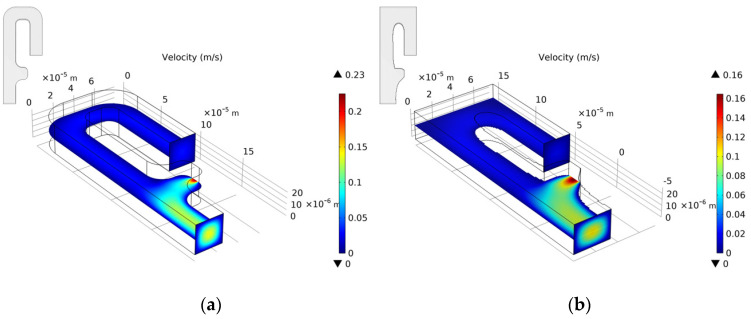
(**a**) The flow field distribution of the optimized structure in [25] and (**b**) the flow field distribution of the optimized structure in this paper of single-cell capture model.

Figure 17 shows the flow field distribution of the structure with the lateral restriction imposed on the capture outlet in [25] and the optimized structure obtained by the topology optimization method in this paper via calculating the flow rate ratio of each outlet and the energy dissipation of the fluid area. The flow rate ratio between the capture channel and the bypass channel is 3.1, and the energy dissipation value is $1.03\times 10^{-5}\;J$. The flow ratio of the capture channel and the bypass channel in the structure obtained by applying the topology optimization method in this paper is 4.8, and the energy dissipation value is $0.431\times 10^{-5}\;J$. It can be seen that compared with the traditional method, the structure obtained by the topology optimization method increases the flow ratio between the capture channel and the bypass channel and reduces the energy dissipation by 58%, which could achieve sequential capture and improve the capture efficiency.

**Figure 18:**
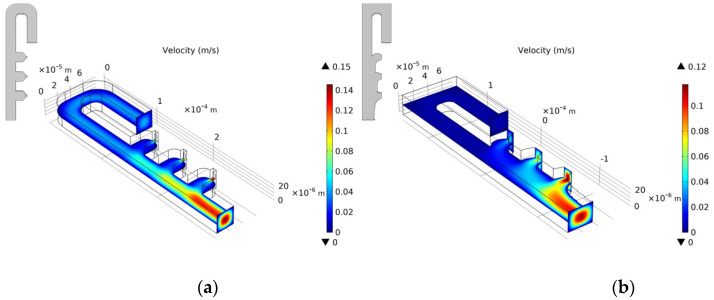
(**a**) The flow field distribution of the optimized structure in [24] and (**b**) the flow field distribution of the optimized structure in this paper of multi-cell capture model.

For the multi-cell capture array, Figure 18 shows the flow field distribution of the optimized structure in [24] and the optimized structure in this paper. The flow ratio of the capture structure in [24] is 0.85:0.65:0.50:1, and the energy dissipation value is $1.88\times 10^{-5}\;J$. The flow ratio of each outlet of the structure optimized by the topology optimization method in this paper is 12.30: 6.87:3.56:1, the flow rate of the previous capture channel is about 1.8 times that of the next capture flow, the flow rate between adjacent capture outlets increased by 54.8%, which can greatly improve the capture efficiency, the energy dissipation value is $0.442\times 10^{-5}\;J$, the energy dissipation value is reduced by 76%. In summary, the topology optimization method of the cell trap discussed in this article is effective.

## 4. Conclusions

This paper discusses the structural design method of the cell traps based on fluid topology optimization. The method is flexible, effective, and can control the flow ratio among the capture channels, the structure topology by increasing or reducing the design area and the fluid flow direction. Under the optimization objective of minimum fluid energy dissipation, single-cell capture and multi-cell capture structures can be achieved. By comparing the effects of changes in multiple parameters on the optimization results, it can provide designers with a reference for parameter selection during the design process. The effectiveness of the design method is verified by comparing the optimized designs with traditional designs in the recently published research literature.

## Nomenclature

**Variable**	**Explanation**	**Characteristic**	**Unit**
$n_{q}$	flow rate ratio between the two outlets in a single capture unit	value related to fluid flow	1
$Q_{\mathrm{trap}}$	flow rate of the capture channel in a single capture unit	-	$m^{3}/s$
$Q_{\mathrm{bypass}}$	flow rate of the bypass channel in a single capture unit	-	$m^{3}/s$
$n_{Q}$	flow rate ratio between the former and latter capture flow channels in a multi-capture unit	-	1
$Q_{j}$	flow rate of the *j*th capture channel in a multi-capture unit	-	$m^{3}/s$
Ω	design area	geometry-related parameter, defined by the designer	$m^{2}$
ρ	fluid density	fluid property parameter	$\mathrm{kg}/m^{3}$
***u***	fluid velocity	value related to fluid flow	m/s
p	fluid pressure	-	Pa
η	fluid dynamic viscosity	fluid property parameter	$N\cdot s/m^{2}$
α	impermeability of the porous medium	solid material property parameter	$m^{2}$
γ	design variable	parameter related to optimization	dimensionless
$\alpha_{\max}$	maximum value of α	parameter related to optimization, a scalar determined by the designer	$m^{2}$
$\alpha_{\min}$	minimum value of α	-	$m^{2}$
q	a positive parameter used to adjust the convexity of the interpolation curve	-	dimensionless
Re	Reynolds number of fluid flow	fluid flow parameter	dimensionless
J	objective function of the optimization model	process value related to optimization	J
Φ	energy dissipation in the computation domain	-	J
$\Phi_{\mathrm{real}}$	energy dissipation of the real flow field	-	J
$\Phi_{\mathrm{ref}}$	energy dissipation of the reference flow field	-	J
$v^{T}\left(1-\gamma \right)$	volume of the solid phase material in the design domain	-	$m^{3}$
b	volume coefficient, used to adjust the appropriate structural volume	adjustable parameter related to optimization, a scalar defined by the designer	dimensionless
θ	weighting coefficient of the flow constraint	-	dimensionless
$\theta_{0}$	initial value of weighting coefficient of the flow constraint	-	dimensionless
$R_{i}^{\left(k\right)}$	relative error at the *i*th outlet flow of the kth step	value related to fluid flow	1
$\Omega_{\mathrm{cell}}$	a circular area reserved for cells	geometry-related parameter, defined by the designer	$m^{2}$
$\Omega_{C}$	bypass channel domain	-	$m^{2}$
$\Omega_{D}$	design domain	-	$m^{2}$
$\Gamma_{\mathrm{in}}$	inlet of the capture unit	-	m
$\Gamma_{\mathrm{out}}$	outlet of the capture unit	-	m
W	inlet width and bypass outlet width	-	m
$W_{\mathrm{trap}}$	capture channel outlet width	-	m
$L_{C0},L_{C1},L_{C2}$	flow channel length	-	m
$\Omega_{W}$	designated partial solid area	-	$m^{2}$
